# Metabolic comparison of minimally to noninvasive urogenital sample types for studying gynecologic health: A pilot study

**DOI:** 10.1016/j.isci.2025.112938

**Published:** 2025-06-18

**Authors:** Holly Chatenoud, Paweł Łaniewski, Nichole D. Mahnert, Melissa M. Herbst-Kralovetz

**Affiliations:** 1 Department of Basic Medical Sciences, College of Medicine – Phoenix, University of Arizona, Phoenix, AZ 85004, USA; 2 Department of Life Sciences, University of Bath, Bath BA2 7AY, UK; 3 Department of Obstetrics and Gynecology, College of Medicine – Phoenix, University of Arizona, Phoenix, AZ 85004, USA

**Keywords:** Women’s health, Microbiome, Metabolomics

## Abstract

In this pilot study, we investigated three minimally to noninvasive biospecimens: cervicovaginal lavages (CVLs), vaginal swabs, and urine, from 18 premenopausal women using untargeted metabolomics to inform future gynecologic research. Metabolic profiles were compared between specimen types and between participants grouped based on vaginal microbiome composition and body mass index (BMI). We identified metabolites altered in the groups in all sample types; however, different metabolites were uniquely detected in each biospecimen. Within CVLs, key amino acid signatures (i.e., polyamines) were identified in women with dysbiosis. While polyamines were also detected in vaginal swabs, this sample type exhibited more lipid signatures. In urine, we specifically detected estrogenic steroids, endocrine-disrupting compounds, and certain drugs. Overall, each urogenital biospecimen type exhibits unique metabolic profiles and, based on metabolic alteration in the context of vaginal microbiome and BMI, can be valuable tools in answering questions related to gynecologic health.

## Highlights

• Each biospecimen detects distinct metabolites, offering complementary insights
• Urine provides unique insights into the systemic macroenvironment
• CVLs provide greater information on vaginal biogenic amines
• Vaginal swabs provide deeper information on vaginal lipid profiles

## Graphical abstract

**Figure:**
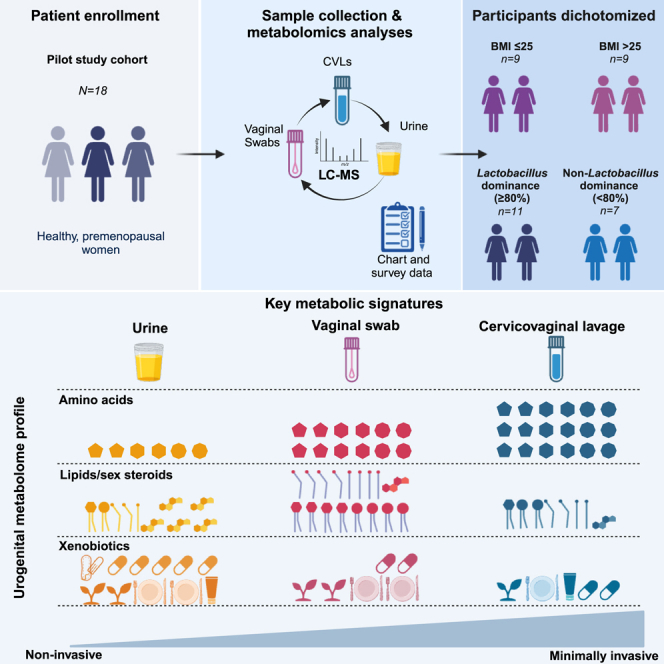

## Introduction

There are numerous technologies used for diagnostic testing in the context of gynecologic health. For detection of benign gynecologic conditions or endometrial cancer, these include transvaginal ultrasound,^1^^,^^2^^,^^3^ laparoscopic surgery,^4^ and hysterectomy.^5^ Yet, the tests used for diagnostics can be invasive, uncomfortable, and may act as a deterrent for screening and diagnosis, leading to worsening gynecologic health effects. This has led to a call for research on sampling methods that are less invasive, more acceptable and accessible, but equivalent to existing methods in terms of predictive accuracy. Different biospecimens have individual benefits and limitations that can influence their use in the clinic and in research. This can depend on the demographics of the population they are collected from, including age,^6^^,^^7^ race/ethnicity,^8^ cultural and belief systems, and geographic location.^9^^,^^10^ These factors can influence the acceptability of urogenital sample types and how widely accessible sampling is within a population.

In this pilot study, we investigated three patient-matched biospecimens: urine, cervicovaginal lavage (CVL), and vaginal swabs. The samples were collected from generally healthy, premenopausal women (*N* = 18) with no previously diagnosed gynecologic conditions. The participants were dichotomized based on their vaginal microbiome composition, and body mass index (BMI). A typically healthy vaginal microbiome is often dominated at the high relative abundance level (80–100%) by one or few protective *Lactobacillus* species, which produce lactic acid and acidify the cervicovaginal microenvironment (pH ≤ 4.5). When vaginal dysbiosis occurs, a depletion of *Lactobacillus* species and an increased growth of dysbiotic anaerobes is observed and elevation of vaginal pH (>4.5).^11^ All of the selected sample types are currently used within both research and clinical settings to assess multiple facets of gynecologic and female reproductive health. These biospecimens are minimally (vaginal swabs and CVLs) to noninvasive (urine) sampling methods. Of three selected biospecimens, only CVLs must be collected by a trained physician during a pelvic exam using a speculum, which may cause anxiety and discomfort to the patient. However, this sampling method is still considered minimally invasive. Introducing the option for self-collection of samples in the clinic or from home can help to address the concern of invasive sampling methods. Vaginal swabs can be performed either by a physician or can be self-collected, and urine samples are commonly self-collected. Generally, self-collected vaginal swabs are considered highly acceptable and cause minimal discomfort.^12^^,^^13^ Urine is a noninvasive, highly accepted sample type that has commonly been used for pregnancy testing, sexually transmitted infection (STI) testing,^14^ and more recently human papillomavirus (HPV) detection.^15^ While all three biospecimens can be used to detect common STI pathogens, such as *Chlamydia trachomatis*, *Neisseria gonorrhoeae,* and herpes simplex virus (HSV),^16^^,^^17^^,^^18^^,^^19^ the Centers for Disease Control and Prevention recommends vaginal swabs or first-void urine to diagnose vaginal *C. trachomatis* infections. Meanwhile, CVLs are mostly used for research studies.

Recent research evaluating the use of these three biospecimens have compared urine to cervicovaginal sampling,^20^^,^^21^^,^^22^ self-collected vaginal swabs to physician-collected CVLs,^23^^,^^24^ and self-collected versus physician-collected urogenital samples^25^^,^^26^^,^^27^ for detection of lower reproductive tract infections. However, unique to our pilot study, we have used a global untargeted metabolomics analysis to illustrate the strengths and limitations of all three patient-matched biospecimen types for research purposes, diagnostic, and/or prognostic testing of gynecological diseases. Our previous studies identified that immunometabolic profiling of CVL samples was predictive for benign gynecologic conditions (e.g., adenomyosis)^28^ and endometrial cancer.^29^ In addition, these analyses were able to provide information on the pathophysiologic mechanisms of these understudied diseases and determine the ability of metabolites detected within CVLs to predict key endometrial tumor characteristics.^28^^,^^29^^,^^30^ The aim of this study was to compare metabolic profiles of each urogenital biospecimen (urine, CVL, and vaginal swab), measured using ultra high-performance liquid chromatography-mass spectrometry (UPLC-MS/MS), and to determine which metabolic markers can be detected in each biospecimen, that could ultimately inform on selection of sampling method for future research studies, applicable to a range of research questions. We hypothesize that we will be able to detect similar metabolic profiles between biospecimens localized to the cervicovaginal mucosa (CVLs and vaginal swabs). However, we would expect a more distinct metabolic profile identified in the urine sample type, allowing for investigation into both localized and systemic metabolic responses. Furthermore, we compared metabolic composition of samples between participants stratified based on vaginal microbiome composition and BMI. In the future, this work could be translated to the clinic to progress noninvasive, accurate sampling and improve gynecologic health outcomes.

## Results

### Patient characteristics

Participants were dichotomized into groups defined by their vaginal *Lactobacillus* dominance (≥80% vs. <80% relative abundance). The *Lactobacillus* dominant (LD) group contained profiles dominated by two *Lactobacillus* spp., mostly *Lactobacillus crispatus* (in 8 participants) or *Lactobacillus iners* (in three participants). Whereas the non-*Lactobacillus* dominant (NLD) group contained profiles with a greater abundance of dysbiotic vaginal bacteria, including *Fannyhessea vaginae*, *Bifidobacterium* spp., and *Prevotella* spp., as well as *L. iners*. The relative abundance of vaginal taxa at species level are shown in Figure S1. We also dichotomized the participants by their BMI, comparing the metabolic profiles of participants with a healthy BMI ≤25 and a BMI >25 indicating overweight/obese.

Participant characteristics are reported in Tables 1 and 2. The mean age of the enrolled participants was 30.67 and did not differ significantly when stratifying based on *Lactobacillus* dominance (*p* = 0.9856) or BMI (*p* = 0.5222). There were no significant differences between race and ethnicity for the LD (*p* = 0.5368, *p* > 0.9999) and BMI (*p* = 0.0824, *p* = 0.5765) groups, respectively. However, we observed that women with NLD had a significantly higher mean BMI compared to women with LD (*p* = 0.0299). Socioeconomic factors such as marital status (*p* = 0.6437, *p* = 0.6199), household income (*p* > 0.9999, *p* = 0.2657), and education level (*p* = 0.5928, *p* = 0.1312) did not differ between LD groups or BMI groups, respectively. Participants who used menstrual pads had a significantly higher vaginal LD compared to those that did not (*p* = 0.0491). Use of other menstrual products including tampons and cervical cups did not differ between the LD groups (Table S2). There were no significant differences in menstrual product usage observed in the BMI groupings (Table S1). Contraceptive usage, douching, use of antibiotics, and level of perceived stress (measured by PSS-10 scores) were also not significantly different between the LD and BMI groups. (Tables S1 and S2).

**Table 1:** Participant characteristics dichotomized by *Lactobacillus* dominance

	All (*n* = 18)	*Lactobacillus* dominance (≥80%) (*n* = 11)	Non-*Lactobacillus* dominance (<80%) (*n* = 7)	*p*-value
**Age (mean (S.D.)) (*n* = 18)**	30.67 (8.26)	30.64 (8.96)	30.71 (7.02)	0.9856
**Race (n (%)) (*n* = 17)**				
White	14 (82.35)	9 (90.00)	5 (71.43)	0.5368
Non-White^a^	3 (17.65)	1 (10.00)	2 (28.57)	
**Ethnicity (n (%)) (*n* = 17)**				
Non-Hispanic	14 (82.35)	9 (81.82)	5 (83.33)	>0.9999
Hispanic	3 (17.65)	2 (18.18)	1 (16.67)	
**BMI (n (%)) (*n* = 18)**				
≤25	9 (50.00)	7 (63.67)	2 (28.57)	0.3348
>25	9 (50.00)	4 (36.36)	5 (71.43)	
**BMI (mean (S.D.)) (*n* = 18)**	32.60 (12.55)	27.63 (7.58)	42.54 (14.46)	**0.0299**
**Vaginal pH (n (%)) (*n* = 18)**				
≤4.5	9 (50.00)	7 (63.67)	2 (28.57)	0.3348
>4.5	9 (50.00)	4 (36.36)	5 (71.43)	
**Marital status (n (%)) (*n* = 17)**				
Single/Divorced	11 (64.71)	7 (70.00)	4 (57.14)	0.6437
Married/Domestic Partner	6 (35.29)	3 (30.00)	3 (42.86)	
**Education (n (%)) (*n* = 17)**				
Some College or lower^b^	5 (29.41)	2 (20.00)	3 (42.86)	0.5928
Associate degree or higher^c^	12 (70.59)	8 (80.00)	4 (57.14)	
**Income (n (%)) (*n* = 13)**				
25,000–75,000	5 (38.46)	3 (37.50)	2 (40.00)	>0.9999
75,000 - >100,000	8 (61.54)	5 (62.50)	3 (60.00)	

*p*-values were calculated using Fisher’s exact test for categorical variables and unpaired t-test for continuous variables. Bold represents a significant *p*-value < 0.05. See also Figure S1 and Table S1.

a Black/African American or East Asian.

b Completed some of a college degree or completed high school.

c Completed an associate degree, College/Bachelor’s degree, or post-graduate degree.

**Table 2:** Participant characteristics dichotomized by BMI

	All (*n* = 18)	BMI (≤25) (*n* = 9)	BMI (>25) (*n* = 9)	*p*-value
**Age (mean (S.D.)) (*n* = 18)**	30.67 (8.50)	29.33 (6.50)	32.00 (10.10)	0.5222
**Race (n (%)) (*n* = 17)**				
White	14 (82.35)	9 (100.00)	5 (62.50)	0.0824
Non-White^a^	3 (17.65)	0 (0.00)	3 (37.50)	
**Ethnicity (n (%)) (*n* = 17)**				
Non-Hispanic	14 (82.35)	8 (88.89)	6 (75.00)	0.5765
Hispanic	3 (17.65)	1 (11.11)	2 (25.00)	
**Vaginal pH (n (%)) (*n* = 18)**				
≤4.5	9 (50.00)	6 (66.67)	3 (33.33)	0.3469
>4.5	9 (50.00)	3 (33.33)	6 (66.67)	
***Lactobacillus* dominance (n (%)) (*n* = 18)**				
Lactobacillus dominant (≥80%)	11 (61.11)	7 (77.78)	4 (44.44)	0.3348
Non-Lactobacillus dominant (<80%)	7 (38.89)	2 (22.22)	5 (55.56)	
**Marital status (n (%)) (*n* = 17)**				
Single/Divorced	11 (64.71)	6 (75.00)	5 (55.56)	0.6199
Married/Domestic Partner	6 (35.29)	2 (25.00)	4 (44.44)	
**Education (n (%)) (*n* = 17)**				
Some College or lower^b^	5 (29.41)	1 (11.11)	4 (50.00)	0.1312
Associate degree or higher^c^	12 (70.59)	8 (88.89)	4 (50.00)	
**Income (n (%)) (*n* = 13)**				
25,000–75,000	5 (38.46)	1 (16.67)	4 (57.14)	0.2657
75,000 - >100,000	8 (61.54)	5 (83.33)	3 (42.86)	

*p*-values were calculated using Fisher’s exact test for categorical variables and unpaired t-test for continuous variables. See also Table S2.

a Black/African American or East Asian.

b Completed some of a college degree or completed high school.

c Completed an associate degree, College/Bachelor’s degree, or post-graduate degree.

### Biospecimens exhibit distinct global metabolomic profiles

**Figure 1:**
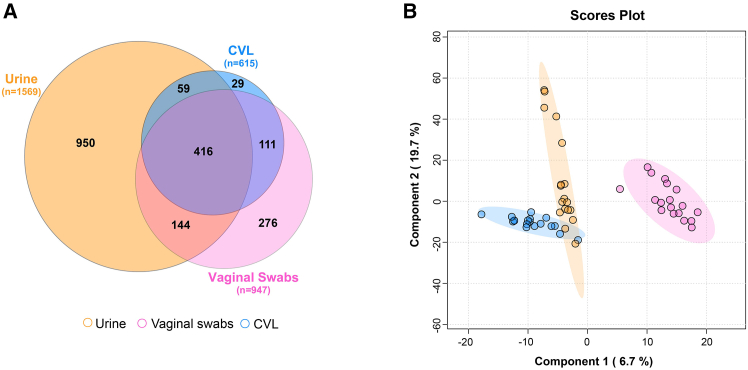
Metabolic profiles differ between biospecimen types (A) A Venn diagram, created using DeepVenn, representing the number of metabolites detected in each biospecimen, and the number of shared and unique metabolites between the sample types. (B) Partial least squares-discriminant analysis (PLS-DA) presenting the variance observed in the metabolome between the three biospecimens. See also Figures S2 and S3.

Metabolic profiles of each sample type were determined using a global untargeted metabolomics platform. The largest number of metabolites (including partially characterized or uncharacterized metabolites) were detected within the urine (*n* = 1569), followed by the vaginal swabs (*n* = 947). The fewest number of metabolites were detected in CVLs (*n* = 615). Of these, we were able to identify a large number of metabolites with a known identity in the urine (*n* = 1016), CVL (*n* = 549), and vaginal swab (*n* = 805) samples. There were many metabolites shared between all sample types (*n* = 416), yet urine had the highest number of unique metabolites of the four biospecimens (*n* = 950). In comparison, the metabolites detected within CVLs and vaginal swabs were mostly shared (Figure 1A). A partial least squares discriminant analysis (PLS-DA) was used as a data reduction method to compare the global metabolic profiles of the three distinct biospecimens. Two components, used to construct the PLS-DA plot, accounted for 19.7% and 6.7% of the variance of the data. A clear separation was observed between the metabolic profiles of all biospecimen types (Figure 1B). In addition, we observed that, in particular, the vaginal swab samples exhibit greater variation in both component 1 and 2 when compared to CVL (variation observed mostly in component 1) or urine samples (variation observed mostly in component 2). Most of the top metabolites contributing to components one and two included cofactors and vitamins (carotene diol, 3′dephosphocoenzyme A and ɣ/β-tocopherol) and lipid (glycerophosphoethanolamines) metabolites and were mostly detected only in the vaginal swab sample type, (Figure S2). In addition, the hierarchical clustering analysis of swab samples, which included two blank control swabs, revealed that metabolic profiles of vaginal swabs differed from controls (Figure S3). This indicates that there is no profound influence of the swab itself on the unique metabolic profiles observed in the vaginal swabs.

### Metabolic composition differs between biospecimens

**Figure 2:**
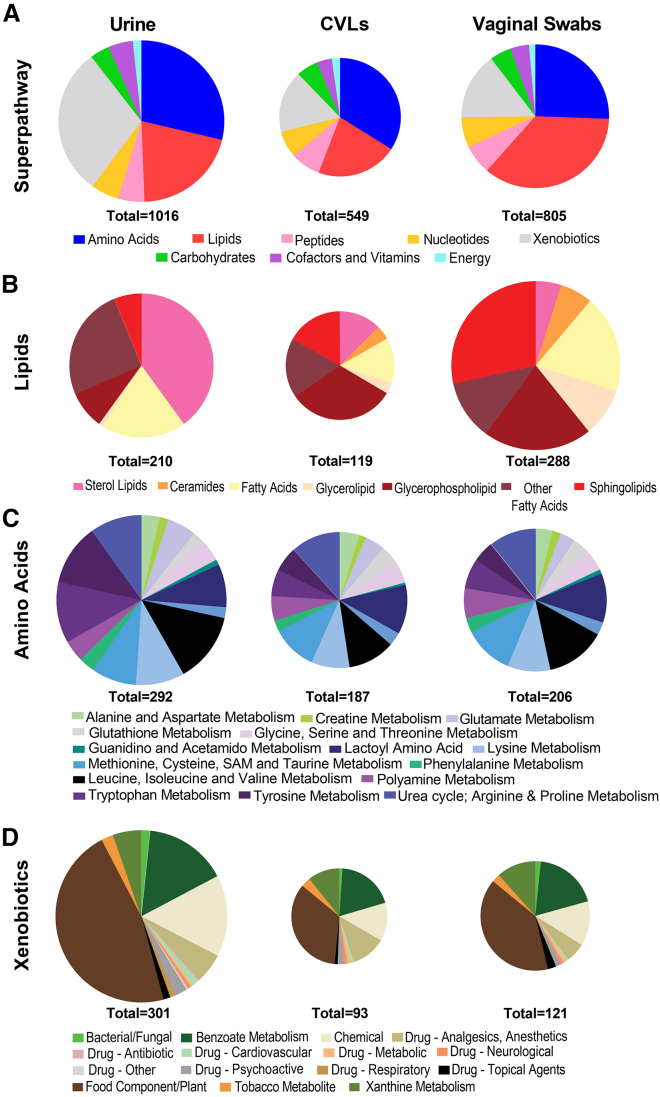
Composition of key superpathways and subpathways differs between biospecimen types Pie charts representing the (A) superpathway, (B) lipid, (C) amino acid, and (D) xenobiotic subpathway compositions in three tested patient-matched biospecimen types. See also Table S3.

Key metabolic superpathway and subpathway compositions, detected in biospecimens reflective of systemic (urine) and local (CVLs and vaginal swabs) microenvironments, were significantly different. When evaluating the differences in superpathway composition we observed that urine was the most unique biospecimen, compared to CVLs (*p* < 0.0001) and vaginal swabs (*p* < 0.0001). The superpathway composition was also significantly different between the CVLs and the vaginal (*p* < 0.0001) swab samples. Three superpathways were most represented in all the biospecimens and included amino acids (18.6%–30.4%), lipids (13.4%–35.8%), and xenobiotics (15.0%–19.2%). However, the distribution of these superpathways differed between the sample types. The largest number of detected metabolites in urine were xenobiotics (*n* = 301), the largest proportion of metabolites detected in CVLs were amino acids (*n* = 187) and the largest number of lipids were identified in vaginal swabs (*n* = 206) (Figure 2A).

Lipid subpathway composition differed the most between the biospecimens. Compared to other specimen types, urine contained many sterol lipids, including estrogenic steroids (which were detected only within urine). The largest proportion of lipids detected in CVLs were glycerophospholipids, whereas more sphingolipids were detected in the vaginal swab samples (Figure 2B). Various amino acid subpathways were detectable in all sample types and the subpathway composition was not significantly different between any of the sample types (Figure 2C).

Xenobiotics were detected in each sample type and included biochemicals related to food, drugs, and chemical exposures. Yet, there were unique xenobiotic signatures to each biospecimen type. Analgesics (acetaminophen), thiazide diuretics (hydrochlorothiazide), diabetes drugs (metformin), neurological (gabapentin), topical drugs (2,6-dihydroxybenzoate) and antidepressants (citalopram and hydroxybupropion) were detected in all sample types. There were no unique xenobiotics detected only in the vaginal swab samples, but this was not the case with other sample types. Urine contained the largest number of uniquely identified xenobiotics, including opioids (oxycodone), antibiotics (doxycycline), tetrahydrocannabinol (THC), antihypertensives (lisinopril, amlodipine, and 4-hydroxycoumarin) and mucolytic drugs (S-carboxymethyl-cysteine). The only drug uniquely detected within CVLs was lidocaine. Metabolites related to chemical exposures were also identified distinctively between biospecimens. Endocrine disrupting compounds (EDCs), such as parabens and phthalates, were both detected in urine, but of those only parabens were also detectable within CVLs. No EDCs were detected within the swab samples (Figure 2D). The number of metabolites belonging to each superpathway and subpathway within each biospecimen are included in Table S3.

### Metabolites identified in urogenital biospecimens are significantly altered in vaginal dysbiosis and elevated BMI

**Figure 3:**
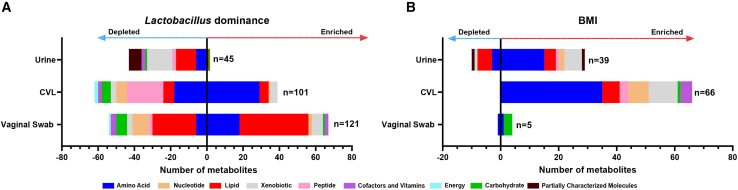
Biospecimens exhibit different superpathway compositions of significantly altered metabolites detected in *Lactobacillus* dominance or BMI groupings Bar plots representing the number of significantly altered metabolites between (A) non-*Lactobacillus* dominant (<80%) and *Lactobacillus*-dominant (≥80%) and (B) healthy BMI (>25) and overweight/obese BMI (≤25) groupings in three urogenital biospecimens. Significantly altered metabolites were determined using differential abundance analyses, using non-parametric Mann-Whitney statistical tests (*p* < 0.05, FC ≥ 2.0 or ≤ -2). The bars are color coded by superpathway. Uncharacterized metabolites were excluded from this analysis. See also Tables S4, S5, and S6.

Participants for this pilot study were dichotomized by vaginal *Lactobacillus* dominance or BMI categories. In the following analyses we have used these groupings to evaluate which types of metabolites can be identified by each sample type. This approach allowed us to make comparisons on the suitability of the sample types themselves, for identifying particular metabolic features in individuals with vaginal *Lactobacillus* dominance and a healthy BMI (≤25). To analyze the biospecimens further we used LD and BMI groups to compare the metabolic signatures of urogenital samples (urine, CVLs and vaginal swabs). Analysis determining the distribution of altered metabolites detected in these urogenital biospecimens revealed significant differences in both the number and superpathway composition of the altered metabolites identified in each specimen. In addition, differences were observed within the same specimen type when dichotomizing the cohort based on *Lactobacillus* dominance (Figure 3A) or BMI (Figure 3B) categories (healthy vs. overweight/obese).

When comparing NLD (<80%) and LD (≥80%) groups, we observed a large number of significantly (*p* < 0.05) altered metabolites detected in each biospecimen (Figure 3A). Vaginal swabs exhibited the largest number of altered metabolites (*n* = 121), followed by CVLs (*n* = 101) and urine (*n* = 45) between the vaginal microbiota groupings. The superpathway composition of the altered metabolites was consistent with the overall composition of metabolites identified previously within each biospecimen. Metabolites belonging to the lipid, amino acid and xenobiotic superpathways were dysregulated the most in the vaginal swab, CVL and urine samples, respectively. CVL and vaginal swab samples showed more similarities in the metabolite alteration at the superpathway level between LD and NLD groups compared to urine samples. In both vaginal samples (CVLs and vaginal swabs) we observed both an elevation and depletion in amino acid and lipid metabolites, whereas in the urine we mostly observed a depletion. A greater number of peptide metabolites (*n* = 20, 19.8%) were also depleted in CVL samples, compared to those depleted in vaginal swab samples (*n* = 2, 1.7%). In addition, we also observed both an enrichment and depletion of nucleotide metabolites in CVL and vaginal swab samples, which were not significantly altered between LD and NLD in the urine samples. Whilst we observed altered xenobiotics in the CVL and vaginal swab biospecimens, we observed the greatest depletion of xenobiotics (*n* = 14, 31.1%) in the urine of participants with NLD profiles compared to LD profiles (Figure 3A).

Biogenic amines (or polyamines) associated with vaginal dysbiosis were significantly elevated in both the CVLs (Table S5) and vaginal swabs (Table S6) of women with NLD profiles. Key metabolites included putrescine (*p* = 0.0005, *q* = 0.0360) (*p* = 0.0049, *q* = 0.1620), cadaverine (*p* = 0.0003, *q* = 0.0360), (*p* = 0.0028, *q* = 0.1257), tyramine (*p* = 0.0015, *q* = 0.0606), (*p* = 0.0012, *q* = 0.1243), and biogenic amine associated metabolites *N*-acetylputrescine (*p* = 0.0028, *q* = 0.0807)(*p* = 0.0556, *q=* 0.3220), *N*-acetylcadaverine (*p* = 0.0005, *q=* 0.0360)(*p* = 0.0012, *q* = 0.1243). No biogenic amines, or their metabolites, were found to be significantly altered in the urine of women with NLD (Table S4).

Whilst we did not observe altered biogenic amines in the urine sample type, we did identify alterations in lipid metabolites associated with vaginal dysbiosis. Hydroxyacylcarnitine lipids, identified in urine, were significantly depleted in the NLD group. These included (R)-3-hydroxybutyrylcarnitine (*p* = 0.0122, *q* = 0.7705), (S)-3-hydroxybutyrylcarnitine (*p* = 0.0268, *q* = 0.7705) and 3-hydroxyhexanoylcarnitine (*p* = 0.0268, *q* = 0.7705) (Table S4). Similarly, in the CVL and vaginal swab samples acylcarnitine lipids, such as octanoylcarnitine (*p* = 0.0173, *q* = 0.1731), (*p* = 0.0086, *q* = 0.1775), hexanoylcarnitine (*p* = 0.0203, *q* = 0.1826), (*p* = 0.0158, *q* = 0.2274), and acetylcarnitine (*p* = 0.0154, *q* = 0.1636), (*p* = 0.0268, *q* = 0.2520), were also significantly depleted in the NLD group (Tables S5 and S6). Other lipids significantly depleted in only the vaginal swab samples of women with NLD included phosphatidylcholine and phosphatidylethanolamine lipids (Table S6).

When dichotomizing by BMI (Figure 3B) we observed the largest number of altered metabolites in CVLs (*n* = 66), followed by urine (*n* = 39) and vaginal swabs (*n* = 5). Amino acids were significantly altered in all three biospecimens. However, lipids and carbohydrates were also altered between individuals with an overweight/obese BMI >25 and healthy ≤25 within each biospecimen. In CVLs we mostly identified an elevation in amino acids involved in histidine metabolism (i.e., 1-methyl-5-imidazoleacetate (*p* = 0.0052, *q* = 0.2051), 1-methyl-5-imidazolelactate (*p* = 0.0062, *q* = 0.2051), formiminoglutamate (*p* = 0.0134, *q* = 0.2125)) and the urea cycle (i.e., dimethylarginine (ADMA + SDMA) (*p* = 0.0040, *q* = 0.1992), N2,N5-diacetylornithine (*p* = 0.0032, *q* = 0.1992), N-acetylcitrulline (*p* = 0.0469, *q* = 0.3014)).

While amino acids were also significantly elevated in urine of participants with an overweight/obese BMI >25, only one amino acid (formiminoglutamate (*p* = 0.0040, *q* = 0.6890)) was also significantly elevated in the CVLs (*p* = 0.0134, *q* = 2125). Differential abundance analyses revealed that most of the amino acids significantly elevated in the urine belonged to the leucine, isoleucine and valine metabolism (i.e., 3-methyl-2-oxovalerate (*p* = 0.0412, *q* = 0.8761), β-hydroxyisovaleroylcarnitine (*p* = 0.0106, *q* = 0.6890)) and lactoyl amino acid subpathways [i.e., *N*-lactoyl valine (*p* = 0.0027, *q* = 0.6890), *N*-lactoyl histidine (*p* = 0.0142, *q* = 0.7624), and *N*-lactoyl leucine (*p* = 0.0244, *q* = 0.8652)].In addition, we observed alterations in the lipid metabolites in the urine biospecimens. These mostly included acylglycine lipids, which were significantly depleted (*p* = 0.0008–0.0056, *q* = 0.3845–0.6890) in the urine of individuals with a BMI >25 (Table S4).

Within the vaginal swabs, metabolites altered between the BMI groups displayed a different composition to those in the urine or CVL samples. Monosaccharides [glucose (*p* = 0.0315, *q* > 0.9999), ribose (*p* = 0.0166, *q* > 0.9999) and a disaccharide [maltose (*p* = 0.0400, *q* > 0.9999)] were only significantly elevated in individuals with an overweight/obese BMI >25 in the vaginal swab samples (Table S6). Amino acid metabolites, including hydantoin-5-propionate (*p* = 0.0314, *q* = 0.8755) (*p* = 0.0209, *q* > 0.9999), were significantly elevated in overweight/obese BMI >25 and hypotaurine (*p* = 0.0379, *q* = 0.8761) (*p* = 0.0309, *q* > 0.9999), was significantly depleted in BMI >25 when measured in both urine and vaginal swab samples, respectively. However, these amino acids were not significantly altered within CVL samples. The fold change, *p*-values and *q*-values of all significantly altered metabolites between BMI and LD groupings are shown in Tables S4, S5, and S6.

### Metabolites identified in urogenital samples are discriminatory for *Lactobacillus* dominance

**Figure 4:**
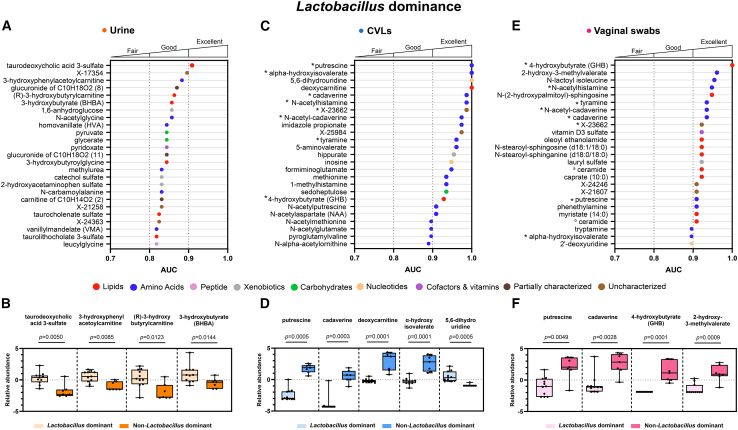
Metabolites detected in urogenital biospecimens are good/excellent discriminators for *Lactobacillus* dominance The top 25 metabolites with the largest area under the curve (AUC) detected within each biospecimen were selected and are shown in (A), (C), and (E). The relative abundance of key metabolites in individuals with *Lactobacillus* dominant (≥80%) and non-*Lactobacillus* dominant (<80%) samples are displayed in (B), (D), and (F). Metabolites are color coded by superpathway. An asterisk represents the same metabolite was a good/excellent discriminator for *Lactobacillus* dominance when detected in more than one biospecimen. ^a^ Ceramide (d18:1/20:0, d16:1/22:0, d20:1/18:0). ^b^ Ceramide (d18:1/17:0, d17:1/18:0). Significant differences in metabolite relative abundances between the groups were calculated using a non-parametric Mann-Whitney test. See also Figure S4.

Univariate receiver operating characteristic (ROC) analyses revealed metabolites detected in all three biospecimen types were able to discriminate between LD and NLD groups, indicating the suitability of all three sample types for use in urogenital microbiome related studies (Figure 4).

The metabolites detected in the urine that were highly discriminatory for LD vs. NLD were not similarly identified with the localized CVL or vaginal swab samples (Figure 4A). Within the urine samples metabolites from numerous metabolic superpathways were good predictors (AUC>0.8) for the LD groups. Secondary bile acids (taurodeoxycholic acid 3-sulfate (*p* = 0.0050, *q* = 0.7705, AUC = 0.9091), taurocholenate sulfate (*p* = 0.0264, *q* = 0.7705, AUC = 0.8247), taurolithocholate 3-sulfate (*p* = 0.0297, *q* = 0.7705, AUC = 0.8182)) were significantly depleted in NLD. Urinary 3-hydroxybutyrate (BHBA) (*p* = 0.0144, *q* = 0.7705, AUC = 0.8636) and acylcarnitine metabolites, (R)-3-hydroxybutyrylcarnitine (*p* = 0.0123, *q* = 0.7705, AUC = 0.8636), and 3-hydroxyphenylacetoylcarnitine (*p* = 0.0085, *q* = 0.7705, AUC = 0.8831) were also significantly depleted in NLD (Figure 4B).

We observed that eight metabolites were excellent discriminators (AUC>0.9) for LD when detected in both CVLs and vaginal swabs. These mostly included amino acid metabolites such as putrescine (*p* = 0.0005, *q* = 0.0360, AUC = 1) (*p* = 0.0049, *q* = 0.1620, AUC = 0.9091), ⍺-hydroxyisovalerate (*p* = 0.0001, *q* = 0.0188, AUC = 1) (*p* = 0.0041, *q* = 0.1465, AUC = 0.8961), cadaverine (*p* *=* 0.0003, *q* = 0.0360, AUC = 0.9870) (*p* = 0.0028, *q* = 0.1257, AUC = 0.9351), *N*-acetylhistamine (*p* = 0.0006, *q* = 0.0385, AUC = 0.9870) (*p* = 0.0008, *q* = 0.1243, AUC = 0.9481), *N*-acetylcadaverine (*p* = 0.0005, *q* = 0.0360, AUC = 0.9740) (*p* = 0.0012, *q* = 0.1243, AUC = 0.9351), and tyramine (*p* = 0.0015, *q* = 0.0606, AUC = 0.9610) (*p* = 0.0012, *q* = 0.1243, AUC = 0.9351). The top 25 metabolites with the highest AUC also included the lipid metabolite, 4-hydroxybutyrate (GHB) (*p* = 0.0005, *q* = 0.0360, AUC = 0.9286) (*p* = 0.0001, *q* = 0.0845, AUC = 1), and the uncharacterized metabolite X-23662 (*p* = 0.0003, *q* = 0.0360, AUC = 0.9870) (*p* = 0.0036, *q* = 0.1335, AUC = 0.9221), which were also excellent discriminators of LD and NLD groups when detected in CVLs and vaginal swabs, respectively. Other vaginal metabolites uniquely identified in CVLs, which were excellent discriminators between the LD and NLD groups, included deoxycarnitine (*p* = 0.0001, *q* = 0.0188, AUC = 1), and 5,6-dihydrouridine (*p* = 0.0005, *q* = 0.0360, AUC = 1) (Figures 4C–4F).

Many lipid metabolites were uniquely identified in vaginal swabs as excellent discriminators for LD (Figure 4E). These included sphingolipids [N-(2-hydroxypalmitoyl)-sphingosine (*p* = 0.0008, *q* = 0.1243, AUC = 0.9481), *N*-stearoyl-sphingosine (*p* = 0.0019, *q* = 0.1243, AUC = 0.9221), *N*-stearoyl-sphinganine (*p* = 0.0019, *q* = 0.1243, AUC = 0.9221)] and ceramides [(ceramide (d18:1/20:2, d16:1/22:0, d20:1/18:0) (*p* = 0.0036, *q* = 0.1335, AUC = 0.9221), ceramide (d18:1/17:0, d17:1/18:0) (*p* = 0.0028, *q* = 0.1257, AUC = 0.9091)], which were significantly enriched in participants with NLD (Figure 4F). Relative abundance data without log-transformation is presented in Figure S4.

Overall, there were numerous metabolites detected within each biospecimen that can be used to discriminate between the NLD group, compared to the LD controls. Metabolites which were highly discriminatory for LD groups were identified in both of the localized biospecimens, CVLs and vaginal swabs. The metabolites identified in urine were not similarly identified as being discriminatory for *Lactobacillus* dominance in the CVL or vaginal swab sample types, hence in this analysis the urine biospecimen exhibited a more unique signature.

### Metabolites identified in urogenital samples are discriminatory for BMI categories

**Figure 5:**
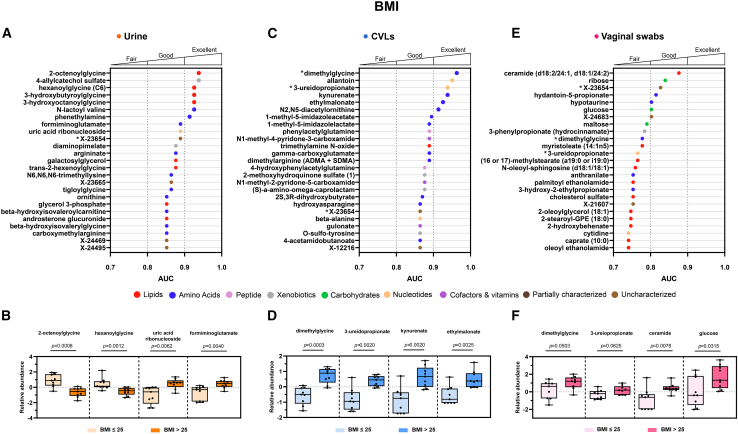
Metabolites detected in urogenital biospecimens are good/excellent discriminators for BMI The top 25 metabolites with the largest area under the curve (AUC) detected within each biospecimen were selected and are shown in (A), (C), and (E). The relative abundance of key metabolites in individuals with a healthy BMI (≤25) and an overweight/obese BMI (>25) are displayed in (B), (D), and (F). Metabolites are color coded by superpathway. An asterisk represents the same metabolite was a good/excellent discriminator for BMI when detected in more than one biospecimen. Significant differences in metabolite relative abundances between the groups were calculated using a non-parametric Mann-Whitney test. See also Figure S5.

There are metabolites detected in all three urogenital biospecimens that elicit a good ability to discriminate between BMI groups (healthy (≤25) vs. overweight/obese (>25)). Metabolites detected within the urine and CVL samples were the best discriminators for BMI (Figures 5A and 5C).

Within the urine biospecimens many lipid metabolites were excellent discriminators for a heathy BMI (≤25), specifically acylglycine lipids (Figure 5A). Of those, 2-octenoylglycine (*p* = 0.0008, *q* = 0.3845, AUC = 0.9383), hexanoylglycine (*p* = 0.0012, *q* = 0.3845, AUC = 0.9259), 3-hydroxybutyroylglycine (*p*= 0.0008, *q* = 0.3845, AUC = 0.9259), and 3-hydroxyoctanoylglycine (*p* = 0.0012, *q* = 0.3845, AUC = 0.9259), were all significantly depleted in participants with a BMI >25 when compared to a BMI ≤25. Other BMI-associated urinary metabolites, uric acid ribonucleoside (*p* = 0.0062, *q* = 0.6890, AUC = 0.8889), 4-allylcatechol sulfate (*p* = 0.0008, *q* = 0.3845, AUC = 0.9383) and formiminoglutamate (*p* = 0.0040, *q* = 0.6890, AUC = 0.8889), were also highly discriminatory and were significantly elevated in individuals with an overweight/obese BMI >25 (Figure 5B).

In the CVL samples, we observed that the top discriminators for BMI are amino acids and nucleotides including dimethylglycine (*p* = 0.0003, *q* = 0.1725, AUC = 0.9630), allantoin (*p* = 0.0014, *q* = 0.1992, AUC = 0.9506), 3-ureidopropionate (*p* = 0.0020, *q* = 0.1992, AUC = 0.9383) kynurenate (*p* = 0.0020, *q* = 0.1992, AUC = 0.9383), ethylmalonate (*p* = 0.0025, *q* = 0.1992, AUC = 0.9259), 1-methyl-5-imidazoleacetate (*p* = 0.0052, *q* = 0.2051, AUC = 0.8951) and 1-methyl-5-imidazolelactate (*p* = 0.0062, *q* = 0.2051, AUC = 0.8889). All these metabolites were significantly elevated in individuals with an overweight/obese BMI >25 (Figure 5D).

Metabolites detected in vaginal swabs were less discriminatory for BMI than those detected in the other two biospecimens. However, selected metabolites still acted as good discriminators between the BMI groups (Figure 5E). These metabolites included a ceramide [d18:2/24:1, d18:1/24:2 (*p* = 0.0078, *q* > 0.9999, AUC = 0.8765)], monosaccharides [ribose (*p* = 0.0166, *q* > 0.9999, AUC = 0.8395), glucose (*p* = 0.0315, *q* > 0.9999, AUC = 0.8025], and amino acid metabolites (hydantoin-5-propionate (*p* = 0.0209, *q* > 0.9999, AUC = 0.8148) and hypotaurine (*p* = 0.0309, *q* > 0.9999, AUC = 0.8025)) (Figure 5F).

Dimethylglycine and 3-ureidopropionate were identified as discriminators for BMI in both CVLs and vaginal swabs. However, we did not observe a significant difference in dimethylglycine (*p* = 0.0503, *q* > 0.9999), and 3-ureiodpropionate (*p* = 0.0625, *q* > 0.9999) between BMI groups when measured in vaginal swabs. Overall, all three biospecimens each reveal a unique metabolic signature, which are characteristic markers of an elevated BMI. Further relative abundance data without log-transformation is presented in Figure S5.

## Discussion

To our knowledge, this is the first study evaluating patient-matched biospecimens: urine, CVLs, and vaginal swabs, utilizing a global untargeted metabolomics analysis to reveal their benefits and limitations in the context of gynecology research. The use of patient-matched samples is valuable strength to our study, allowing for better comparison between individuals and sample types. Our study revealed that each biospecimen type is unique and multi-faceted in terms of detection of specific types of metabolites, invasiveness level, acceptability, and feasibility. Other factors including personnel required for collection and amount of material that can be extracted for downstream assays can also influence what sample type may be optimal to use depending on the research question or clinical application at hand. Urine and vaginal swabs can both be self-collected, whereas CVLs must currently be collected by a trained physician within the clinic; however, cervical secretions can be self-collected and are being utilized in other studies.^31^^,^^32^ The amount of material that can be extracted from each sample type for downstream analysis is also different. A larger amount of material can be collected with urine and CVLs, as larger volumes of these liquid-based sample types were collected and could be aliquoted for downstream analyses. However, less material can be extracted from the vaginal swabs as these were collected and stored as dry swabs. In our analysis the entire swab was extracted for the metabolomics analysis.

The method by which the samples are collected and pre- and post-processed may also influence the metabolic signature that can be identified in each biospecimen. For example, lipid composition differed the most between the urine, CVL and swab biospecimens. Our study revealed that more lipid metabolites, specifically sphingolipids, were measured in the vaginal swab samples. Sphingolipids are membrane associated lipids involved in epithelial barrier function.^33^^,^^34^^,^^35^^,^^36^ The higher detection of sphingolipids in the swab sample type could result from collection of epithelial cells by swabbing mucosal surfaces. Furthermore, in contrast to the liquid-based CVL and urine samples, the swab biospecimens were not processed by centrifugation to remove cellular debris, which could explain the higher detection of cellular lipids within the swab samples.

Global untargeted metabolomics analysis revealed that metabolites belonging to the amino acid, lipid and xenobiotic superpathways were the most abundant in all four biospecimens. However, each sample type elicited distinct differences in overall metabolic composition. One commonly altered group of metabolites across tested biospecimens were biogenic amines (BAs). Biogenic amines are well established metabolites associated with bacterial vaginosis (BV).^37^^,^^38^ By detecting BAs, we can predict a dysbiotic vaginal microenvironment, which can play a role in gynecologic infection and disease. Key BAs associated with vaginal dysbiosis include putrescine, cadaverine, tyramine and spermidine, as well as precursor amino acids: ornithine and arginine.^37^^,^^39^ Putrescine and cadaverine are two key metabolites that contribute to vaginal malodor, one of the clinical manifestations of BV.^40^ These amines and their precursors were detectable within all four biospecimens but were only significantly associated with NLD profiles within the CVL and vaginal swab samples, not the urine.

Our pilot study also revealed other metabolites associated with vaginal dysbiosis, including lipid metabolites: glycerophospholipids and sphingolipids. Sphingolipids and ceramides were excellent discriminators for NLD when measured in vaginal swabs in our cohort. Sphingolipids have previously been associated with inflammation, including genital inflammation,^41^^,^^42^^,^^43^ and cell death^44^ indicating their importance in the urogenital microenvironment. In addition, glycerolipids and sphingolipids have been elevated in *in vitro* cervical cell cultures after infection of with dysbiotic vaginal microbes.^45^ However, further research is required to determine their association with vaginal dysbiosis. Glycerophospholipids, measured in CVL and vaginal swab samples, were significantly depleted in NLD. This is in accordance with our previous clinical study conducted using CVL samples in which a depletion of glycerophospholipids (glycerophosphoglycerol, glycerophosphoethanolamine, glycerophosphorylcholine, and glycerophosphoserine) was predictive of the elevated vaginal pH (pH > 4.5) and NLD groups.^41^

Acyl-carnitine metabolites were also significantly depleted in NLD when identified in all three urogenital sample types, but only urinary 3-hydroxyphenylacetoylcarnitine and (R)-3-hydroxybutyrylcarnitine were highly discriminatory between the LD groups. This is in agreement with previous studies in which acyl-carnitine metabolites were significantly positively associated with genital inflammation, yet also with *Lactobacillus* abundance.^39^^,^^43^ Urinary secondary bile acids (taurodeoxycholic acid 3-sulfate, taurocholenate sulfate and taurolithocholate 3-sulfate) were also significantly depleted in NLD. Whilst previous studies have investigated the potential mechanisms of intestinal *Lactobacillus* spp. to resist toxicity from bile acids,^46^ there have been no studies associating bile acid excretion to vaginal *Lactobacillus* spp. Further investigation is required to explain the association between urinary bile acids and cervicovaginal health.

An elevated BMI is a known factor that can influence the vaginal microbiome.^47^^,^^48^ Hence in this study we also wanted to investigate if differences in metabolites were alterated between BMI groups (healthy vs. overweight/obese) in each urogenital biospecimen. Within the urine samples, we observed a depletion in acylglycine lipids to be highly discriminatory for BMI. Previous metabolomics studies have identified lower urinary excretion of acylglycines in participants with an elevated BMI, compared to their lean controls.^49^ In the CVL samples we observed that amino acid metabolites (i.e., kynurenate and ethylmalonate) previously associated with increased BMIs were also highly discriminatory for an overweight/obese BMI in our study.^50^^,^^51^ Dimethylglycine and 3-ueidopropionate were identified as discriminators for BMI when detected in both CVL and vaginal swab samples. Dimethylglycine is a metabolite of glycine, hence an increase in urinary dimethylglycine excretion reflects a decrease in circulating glycine, which is a marker of an elevated BMI.^49^ However, this association has not previously been explored in the context of cervicovaginal microenvironment. Three-ureidopropionate (also known as *N*-carbamoyl-β-alanine) has also been associated with a higher BMI.^52^ However, other studies observed a depletion of *N*-carbamoyl-β-alanine in type 2 diabetes mellitus, resulting in conflicting results.^53^ Unique to the vaginal swab samples we observed an elevation in metabolites associated with an overweight/obese BMI including ceramide (d18:2/24:1, d18:1/24:2), and the monosaccharide glucose and that was an excellent discriminator for BMI >25. Studies investigating ceramide content in skeletal muscle revealed an elevation of intramyocellular ceramides in individuals with an elevated BMI.^54^^,^^55^ A higher BMI is also often associated with insulin resistance and therefore higher concentrations of circulating glucose.^56^ Increased vaginal glucose is also thought to be due to the vaginal *Lactobacillus* spp. metabolizing glycogen within vaginal fluid into glucose and maltose; however, this association has not been investigated in the context of women with an elevated BMI.^47^

Xenobiotics, which are exogenous metabolites not produced by the host organism,^57^ were identified in all three biospecimens. These compounds originate from food, drugs, and environmental exposures. Urine contained the greatest number of xenobiotics, which included drug-related metabolites and chemical exposures. We also observed key similarities and differences in which drugs can be detected in each biospecimen. Drugs can influence the vaginal microbiome and in turn affect female sexual and gynecologic health. Therefore, detection of drugs in the urogenital microenvironment can help to better understand potential mechanisms influencing adverse gynecologic health outcomes. For example, use of antidepressants can result in side effects of sexual dysfunction and reduction in libido in some women,^58^^,^^59^ potentially impacting women’s quality of life. In our study citalopram (a common antidepressant) was detectable in all the biospecimen types. Another commonly used class of drugs are nonsteroidal anti-inflammatory drugs (NSAIDs), such as ibuprofen and aspirin. Ibuprofen was also detected in all biospecimen types, but aspirin was uniquely detected within urine samples. Aspirin is cleared via the kidneys and therefore excreted in the urine^60^ which is reflected within our study. Lidocaine was exclusively identified in one participant’s CVL sample. However, we did not detect the parent drug lidocaine (or it is metabolites) in the patient-matched vaginal swab or urine samples from the same participant. Topical anesthetics, including lidocaine and benzocaine, is easily accessible through over the counter (OTC) vaginally applied products, including pain relief sprays for postpartum,^61^ and lidocaine gels for general vaginal pain and itching.^62^^,^^63^^,^^64^

Estrogens, progestogens, and androgens are the three types of sex hormones that are important for regular function of the female reproductive tract.^65^ Androgenic steroids and pregnenolone steroids (such as dehydroepiandrosterone sulfate (DHEA-S), androstenediol (3β,17β) disulfate, and pregnen-diol disulfate) were detectable in all biospecimen types, with the largest number being detected within urine. Estrogenic steroids (estriol 3-sulfate, estriol 16-glucuronide) and progestin steroids (5⍺-pregnan-3β,20⍺-diol disulfate, 5⍺-pregnan-diol disulfate, pregnanediol-3-glucuronide) were only detected within the urine samples. Steroids are lipophilic molecules; thus, to be excreted, they must be metabolized in the liver to become hydrophilic. In the liver, steroids are sulfonated and glucuronidated and can then be excreted via two routes, through the kidney or biliary systems, resulting in excretion through the urine or feces.^66^

Endocrine disrupting compounds (EDCs) are synthetic and natural chemicals, which can interfere with hormones in the endocrine system.^67^ Sources of exposure include personal care and menstrual products, which are disproportionately used by women.^68^^,^^69^^,^^70^^,^^71^^,^^72^ Two EDCs frequently found in personal care products include parabens and phthalates, which were both detected in urine samples in our cohort. Whilst exposure to parabens and phthalates can result from vaginally applied products, including menstrual products^73^ and lubricants^74^; phthalates were undetectable in the CVL and vaginal swab samples. However, parabens were detected in both the urine and CVL samples. Similarly to sex hormones, phthalates are metabolized by undergoing conjugation in the liver. In our study, we only detected glucuronide conjugates of the phthalate metabolites. Hepatic conjugation of phthalates results in excretion via the urine or feces through urinary or biliary excretion, respectively.^75^ Importantly, EDCs can affect women’s health outcomes and have been associated with gynecologic and obstetric conditions including uterine fibroids,^76^^,^^77^^,^^78^ endometriosis,^76^^,^^77^^,^^79^ polycystic ovary syndrome (PCOS),^76^^,^^77^ and pre-term birth^79^; therefore, urine and CVL sampling can be utilized to study exposures to EDCs in the context of the urogenital health.

Metabolic profiles can differ greatly depending on the disease status of the participants being studied. However, as our study was performed on participants without disease, it provides an unbiased overall comparison of the ability of the sample types themselves to detect metabolites. A previous metabolomics study on CVL samples, collected from women with and without bacterial vaginosis (BV), was able to identify 279 metabolites, which is much lower than the number identified within our study. However, similarly to our study Srinivasan, S *et al*.^39^ observed a greater alteration of amino acids in women with BV, reflective of the altered amino acid signature we observed in participants with NLD vaginal profiles. In addition, another study using vaginal swabs was also able to identify fewer metabolites than detected in our study; however, they were able to identify high levels of 4-hydroxybutyrate (GHB) were also indicative of a dysbiotic vaginal profile,^80^ similarly to the findings our study. These studies exploring the cervicovaginal metabolome, identified in CVL and vaginal swab samples, were measured using a combination of gas chromatography-mass spectrometry (GC-MS) and liquid chromatography-mass spectrometry (LC-MS), which could explain the reduced number of metabolites identified compared to our study that used UPLC-MS/MS. Other studies investigating the urinary metabolome also identified fewer metabolites than we did in our study, despite Wang, R et al.^81^ also utilizing UPLC-MS, and Wittmann, B et al.^82^ using LC-MS. However, the latter study only measured amine and lipid profiles which could explain the reduced number of metabolites observed. However, one study also using UPLC-MS/MS, reported a similar number of metabolites in urine to those detected in our study, including drug metabolites such as antidepressants (i.e., escitalopram) and analgesics (acetaminophen and ibuprofen). However, endocrine disruptors and estrogenic steroids were not identified in that study.^82^

**Figure 6:**
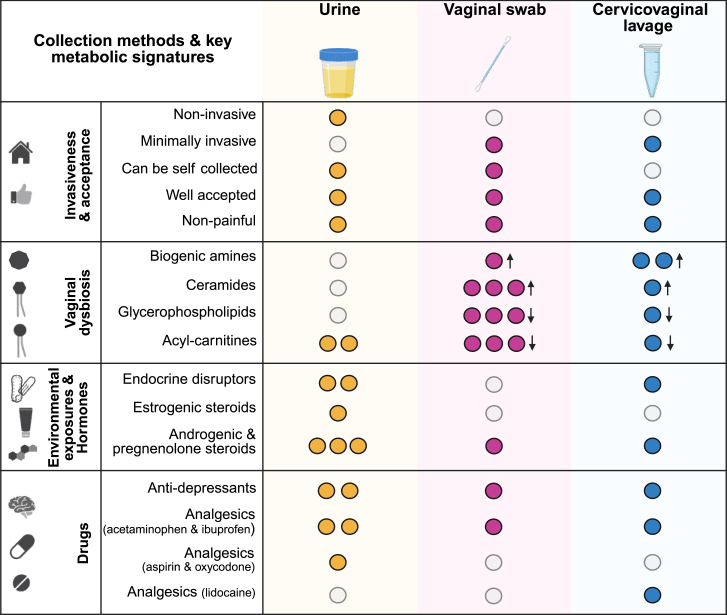
Summary graphic outlining invasiveness and acceptance of tested sample types and detection of key metabolites in three urogenital biospecimens An arrow up indicates a significant (*p* < 0.05, FC ≥ 2.0) enrichment of biogenic amines and ceramides in non-*Lactobacillus* dominant profiles (NLD) (<80% relative abundance). An arrow down indicates a significant depletion (*p* < 0.05, FC ≤ -2) of glycerophospholipids and acyl-carnitine metabolites non-*Lactobacillus* dominant profiles (NLD) (<80% relative abundance).

In summary, through metabolic profiling of three patient-matched biospecimens (urine, CVLs, and vaginal swabs), we found that each biospecimen has benefits and limitations, and can alone or in combination serve as a valuable tool in answering questions related to gynecologic health. In our cohort of generally healthy women, urine provides unique insights into the systemic macroenvironment, including metabolism of hormones, drugs, and environmental exposures. Although CVLs and vaginal swabs exhibited overall different metabolic profiles from each other, both sample types were able to provide unique metabolic information on the localized cervicovaginal microenvironment. with the CVLs providing greater information on biogenic amines such as putrescine and cadaverine, and the vaginal swabs indicating greater sphingolipid signatures (Figure 6). In the context of gynecologic disease, the urogenital metabolic profiles may be altered, thereby potentially altering the metabolic profiles detected within each sample type. Gynecologic health is complex and each biospecimen exhibits a unique metabolic profile. No singular biospecimen can provide insights to all the key metabolic alterations associated with gynecologic disease. However, by combining the use of patient-matched biospecimens, we can gain a better understanding of the functions and interactions of the axes involved in the studied condition. Overall, these biospecimens can inform us on the urogenital microenvironment and this knowledge can be applied to improve women’s health outcomes.

### Limitations of the study

Although our study displays highly significant findings, it is a pilot study, hence, a limiting factor is a small sample size of our cohort. Demographically, our cohort mostly consisted of non-Hispanic, White participants, which is not reflective of the general population. Hence, further studies completed on a larger, more diverse cohort would be beneficial. We did not include survey data evaluating the acceptance of each biospecimen from our participants. Future studies combining survey data with “omics” analyses would reveal greater insights on both the benefits and limitations of biospecimens for clinicians and researchers, but also on comfort and uptake of testing from the participants. Additionally, we acknowledge that we did not adjust for confounding variables between BMI and the vaginal microbiome. Due to a small sample size, we did not have the statistical power to perform these adjustments. However, in larger studies, it may be important to further evaluate associations between the BMI and vaginal microbiome and adjust for confounding variables. Our study also had many strengths, including the use of global metabolomics profiling of patient-matched biospecimens. To our knowledge, this is the only study comparing patient-matched urine, CVL, and vaginal swab samples using global metabolomics analyses. We were able to detect a large number of metabolites with a known identity, ranging from 549 to 1016 metabolites across tested biospecimen types. Some uncharacterized metabolites were also detected, accounting for 10%, 31%, and 14% of all the metabolites identified within CVLs, urine and vaginal swab sample types, respectively. The greatest number of metabolites with unknown identity were measured in the urine samples. This may be a result of the urine reflecting systemic metabolic signatures, in addition to the largest number of metabolites overall being identified within the urine samples. As the identity of these metabolites are unknown, we were unable to analyze these compounds in the context of their function. However, there is a potential for further research into these unknown biochemicals as they become characterized.

## Resource availability

### Lead contact

Requests for further information should be directed to and will be fulfilled by the lead contact, Dr. Melissa M. Herbst-Kralovetz (mherbst1@arizona.edu).

### Materials availability

This study did not generate new unique reagents.

### Data and code availability

#### Data

• Raw spectral data and the original untargeted global LC-MS/MS metabolomics data obtained from Metabolon, Inc. (Morrisville, NC) for the de-identified participants have been depositied at ReData: https://doi.org/10.25422/azu.data.28946582 and are publicly available as of the date of publication.
• The 16s rRNA gene amplicon sequences have been deposited in the National Center for Biotechnology Information (NCBI) database on NCBI BioProject: PRJNA1193618, and is publicly available as of the date of publication at NCBI BioProject: https://www.ncbi.nlm.nih.gov/bioproject/PRJNA1193618.

#### Code

• This paper does not report original code.

#### Additional information

• Any additional information required to reanalyze the data reported in this paper is available from the lead contact upon request.

## Acknowledgments

This study was supported by the 10.13039/100001109Flinn Foundation and the Tom and Catherine Culley Charitable Trust Translational Seed Grant (#22-06544 to M.M.H.-K.). We would like to thank the participants who enrolled in this study and acknowledge Dr. Nicole Jimenez for providing us with her expertise on the vaginal microbiome analyses for this manuscript. In addition P.L. was supported by the Guiding U54 Investigator Development to Sustainability (GUIDeS) program under the award for the Partnership of Native American Cancer Prevention funded by the NIH National Cancer Institute (U54CA143924).

## Author contributions

M.M.H.-K. designed and supervised the study, coordinated metabolomics analysis and funding acquisition. N.D.M. was involved in participant recruitment, sample and clinical data collection. P.Ł. was responsible for sample processing. H.C., P.L., and M.M.H.-K. completed literature searches, analyzed, and interpreted the data, drafted and created the visualization of the data for the manuscript. All authors critically reviewed and approved the final version of the paper.

## Declaration of interests

M.M.H.-K. serves on the scientific advisory board for Freya Biosciences. None of the other authors declare competing interests.

## STAR★Methods

### Key resources table

**Table:** 

REAGENT or RESOURCE	SOURCE	IDENTIFIER
**Biological samples**		

Human urine samples	Banner – University Medicine Women’s Institute, Phoenix, AZ, USA	N/A
Human cervicovaginal lavage samples	Banner – University Medicine Women’s Institute, Phoenix, AZ, USA	N/A
Human vaginal swab samples	Banner – University Medicine Women’s Institute, Phoenix, AZ, USA	N/A

**Chemicals, peptides, and recombinant proteins**		

0.9% saline solution	Teknova, Hollister, CA	Cat#S0699

**Critical commercial assays**		

DNeasy PowerSoil Pro Kit	Qiagen	Cat#47014; RRID: SCR_008539

**Deposited data**		
Raw and processed metabolomics data	Deposited into ReData database	ReData: https://doi.org/10.25422/azu.data.28946582
16s rRNA gene amplicon sequences	Deposited into NCBI database on BioProject	NCBI BioProject: PRJNA1193618; Publicly available at NCBI BioProject: https://www.ncbi.nlm.nih.gov/bioproject/PRJNA1193618; RRID:SCR_004801

**Software and algorithms**		
Metabolon, Inc (global metabolomics platform)	Metabolon, Inc, Durham, NC	metabolon.com
MetaboAnalyst 6.0	MetaboAnalyst	metaboanalyst.ca; RRID:SCR_015539
MicrobiomeAnalyst 2.0	MicrobiomeAnalyst	Microbiomeanalyst.ca; RRID:SCR_015022
BLAST, blastn suite	NIH, blastn suite	https://blast.ncbi.nlm.nih.gov/Blast.cgi; RRID:SCR_004870

**Other**		
FLOQ Nylon-Flocked Dry Swabs in Peel Pouches	COPAN Diagnostics, Murrieta, CA	Cat#502CS01
Micro Essential Lab Single-Roll Hydrion™ pH Test Paper, With Chart; pH range: 4.5 to 7.5	Micro Essential Laboratory Inc, Brooklyn, NY	Cat#334

### Experimental model and study participant details

#### Ethics approval

This study was approved by the Institutional Review Board at the University of Arizona (IRB no. 1806644882) and conducted according to the federal guidelines and the Declaration of Helsinki. Written informed consent was obtained from all participants.

#### Human participant recruitment

Eighteen participants were recruited at the Women’s Institute (Banner University Medical Center, Phoenix, AZ) during their well woman exam between July 2018 and December 2021. Participants were dichotomized to BMI >25 group (*n*=9) or BMI ≤25 group (*n*=9). Additionally, participants were dichotomized as having a non-*Lactobacillus* dominant (<80% relative abundance) (*n*=7) or a *Lactobacillus*-dominant (>80% relative abundance) (*n*=11) vaginal microbiome. We included individuals of all races/ethnicities and socioeconomic statuses who were assigned female at birth, premenopausal, 18 years or older with no diagnosed gynecological conditions. Participants were excluded from the study if they were pregnant; lactating; postmenopausal; currently menstruating; HIV positive; diagnosed with inflammatory bowel disease; currently taking a proton pump inhibitor; experiencing a genital herpes (HSV) outbreak; diagnosed with (or currently being treated for) chlamydia, gonorrhea, trichomonas, or bacterial vaginosis in the past 2 weeks; had douched or used vaginal suppositories or creams in the past 48 hours; had unprotected sexual intercourse in the 24 hours prior to visiting the clinic; had a known infection with *Clostridiodes difficile* colitis; have had chronic antibiotic use (for 2-3 months) or have had treatment for an infection with antibiotics in the past 2 weeks; were on a liquid diet or had used laxatives within the past 7 days prior to biospecimen collection.

#### Patient-related data and sample collection

Patient information on age, weight, height, BMI, comorbidities, allergies, medications, and surgical history were collected from the participants’ medical records. Additional patient-related data, including race, ethnicity, household income, education level, marital status, contraceptive and menstrual product use, recent antibiotics use, was collected using self-reported surveys. A previously validated Perceived Stress Scale (PSS-10)^83^ survey was also utilized in this study. Cervicovaginal lavages (CVLs), two vaginal swabs and two rectal swabs were collected by a physician during a well woman exam. Urine was self-collected by the participants in the clinic. Floqswabs (COPAN Diagnostics, Murrieta, CA) were used for the vaginal swab collections. The vaginal swabs were collected during a routine pelvic exam, after insertion of the speculum. A swab was placed into the posterior vaginal fornix and was rotated for 5 seconds. The first swab was used to determine the vaginal pH using qualitative analysis from observing a color change on Hydrion pH indicator paper 4.5-7.5 (Micro Essential Laboratory Inc, Brooklyn, NY). A second swab was collected using the same method. The vaginal microbiome was sequenced using a vaginal swab. A cervicovaginal lavage was then collected using a sterile 0.9% saline solution. Following collection, all biospecimens were immediately placed on ice and transferred to a -80°C freezer for storage. Prior downstream analyses, the CVL and urine samples were processed. Samples were thawed on ice and centrifuged at 700 × *g* for 10 minutes at 4°C. The supernatant was then aliquoted to prevent multiple freeze-thaw cycles, and frozen -80°C.

### Method details

#### Quantification of soluble metabolites

Untargeted global metabolomics analysis on the three patient-matched biospecimens (urine, CVLs, and vaginal swabs) was performed by Metabolon Inc. (Durham, NC). Metabolites were detected using ultra high-performance liquid chromatography-mass spectroscopy (UPLC-MS/MS). Prior to UPLC-MS, the samples were precipitated with methanol and shaken vigorously (Glen Mills Genogrinder 2000) then centrifuged to remove proteins which could associate with small molecules. This extract was split into five aliquots, one for each analysis and one to spare.

A Waters Acquity UPLC and a Thermo Scientific Q-Exactive high resolution-accurate mass spectrometer, interfaced with a heated electrospray ionization (HESI-II) source and Orbitrap mass analyzer operated at 35,000 mass resolution was used. The samples were then dried and reconstituted into acidic or basic solvents to achieve a fixed concentration to ensure chromatographic consistency. These aliquots were analyzed using acidic, positive ion-optimized and basic, negative ion-optimized conditions in two separate columns (Waters UPLC BEH C18-2.1x100mm, 1.7 μm). The acidic aliquot was gradient eluted using water and methanol containing 0.1% formic acid. The basic aliquot used water and methanol with 6.5 mM ammonium bicarbonate. A third aliquot was analyzed using negative ionization, which was eluted from a HILIC column (Waters UPLC BEH Amide 2.1x150 mm, 1.7μm) using a gradient of water and acetonitrile with 10mM ammonium formate. The metabolites were identified via peak analysis, performed by the Metabolon’s Laboratory Information Management Systems (LIMS). Identified compounds then underwent quality control (QC) procedures. During the QC stage, peaks that represent system artifacts, mis-assignments, mis-integration and background noise are removed or corrected for. Deviations from consistent peak identification from tested samples are examined using proprietary visualization and interpretation software. Peaks which pass the QC checks then go on to further pre-processing steps. Peaks were quantified using area-under-the-curve analysis to measure relative intensity. After determining the raw peak area values, the data was batch-normalized by Metabolon Inc, which is a common normalization method for metabolomics data. This allowed for correction of any differences between peak area intensities between batches. To batch-normalize the data, the individual raw values of each metabolite identified within a sample was divided by the median value of all the metabolites identified in the sample within a particular batch, giving each metabolite a median of one. From here, to futher remove batch variability for each metabolite, each individual datapoint was normalized proportionally to this median of one. For each metabolite the minimum value across all batches was imputed for any missing values, giving us batch-normalized imputed data which was used for downstream analyses. The batch-normalised and imputed data was was then further log_10_-transformed and autoscaled (mean-centered and divided by the standard deviation of each variable) before performing the analyses to ensure a normal distribution of the data.

#### 16S rRNA gene amplicon sequencing

DNA extraction was performed using the DNeasy PowerSoil Pro Kit (Qiagen), as per the manufacturer’s instructions. After DNA extraction, the DNA from the clinician-collected vaginal swabs underwent 16s rRNA sequencing. The 16s rRNA gene was amplified using the V4 hypervariable region, with the primers 515F (GTGCCAGCMGCCGCGGTAA) and 806-R (GGACTACHVGGGTWTCTAAT).^84^ The primers included Illumina adapters and reverse primers included a 12-base pairs (BP) barcode which is unique to each sample. PCR products were then cleaned with QIAseq beads (Qiagen, Hilden, Germany), and quantified using the QUANT-iT PicoGreen dsDNA Assay Kit (Invitrogen, Waltham, MA, USA). Pooled purified DNA products were sequenced on a 2 x 150 bp Illumina Nextseq 1000 platform (Illumina, San Diego, CA, USA) at the PANDA Core for Genomics and Microbiome Research, University of Arizona, USA. Demultiplexed fastq files were then processed using the DADA2 pipeline.^85^ The first 10 and last 5 bp were trimmed. Reads exceeding a maximum expected error of two or more bp were removed. The resulting reads were used to infer amplicon sequence variants (ASVs). Taxonomic identities were associated to each ASV using the RDP classifier.^86^ ASVs were trained on the GTDB version 214 database.^87^ We removed any ASVs with unknown taxonomy at kingdom level. Extraction blanks were used to determine potential contaminants, which were removed using the R package “decontam”.^88^ We removed samples with less than 100,000 reads, leaving a total of 205 samples of Bacteria/Archaea with a mean of 286,246 (+- 97346) reads per sample. Following these steps, the National Center for Biotechnology Information through the National Library of Medicine was utilized for the Nucleotide Basic Local Alignment Search Tool (nBLAST) to further refine the top 100 most abundant vaginal ASVs at the species level.^89^ FASTA sequences were uploaded and searched on rRNA/ITS databases (16S ribosomal RNA sequences (Bacteria and Archaea)) and optimized for somewhat similar sequences (blastn). Following the DNA sequencing, the data was organized into three separate files using Excel, consisting of a metadata file for the participants, a raw counts file identified to the amplicon sequence variant (ASV) level, and a taxonomy table which included the corresponding bacterial taxonomy for each ASV. The three files were uploaded into MicrobiomeAnalyst 2.0,^90^ in which relative abundance analysis was performed. The relative abundance of the vaginal bacteria were calculated at the genus level. Vaginal swab samples with a relative abundance of *Lactobacillus* spp. Of ≤80% were considered Lactobacillus dominant vaginal profiles, samples with <80% *Lactobacillus* relative abundance were considered non-*Lactobacillus* dominant profiles. The vaginal microbiome data can be accessed via the following link: https://www.ncbi.nlm.nih.gov/bioproject/PRJNA1193618.

#### Partial least squares discriminant analysis (PLSDA)

Partial least squares discriminant analysis (PLSDA) was performed using MetaboAnalyst 6.0^91^ to determine the variance between the three biospecimen types. PLSDA plots are used to reduce the dimensionality of the data and allow visualization of the separation of clusters of metabolites within the data. A PLSDA is a supervised data regression method taking into account the assigned groupings to better plot the greatest separation in the data.

#### Differential abundance analysis

Differential abundance analyses are a summative analysis of a two-sample t-test and fold change (FC) analysis. The FC analysis compared the absolute values of change between the means of the metabolites within the two groups. The batch-normalized data was log-transformed and auto-scaled prior to FC analyses to ensure for normal distribution of the data. Hence, T-tests were completed, and *p*-values were reported. Significant metabolites had both a *p*-value <0.05 and a FC ≥2.0 or ≤-2. This analysis was performed using MetaboAnalyst 6.0^91^ to display significantly altered metabolites in individuals with an overweight/obese BMI (>25) and individuals with non-*Lactobacillus* dominant vaginal profiles (<80%) relative to appropriate control groups.

#### Receiver operating characteristic (ROC) analysis

ROC analysis was performed using MetaboAnalyst,^91^ and was used to determine sensitivity and specificity of metabolites to accurately discriminate between the groups. The strength of the metabolites as discriminators for BMI and *Lactobacillus* dominance groups was measured with area under the curve (AUC) values. Metabolites with an AUC >0.9 were considered excellent discriminators.

### Quantification and statistical analysis

Prior to all statistical analyses, the batch-normalized metabolic data was further log_10_ transformed and auto-scaled. Statistically significant differences were represented by a *p*-value less than 0.05. Statistical analyses were performed using MetaboAnalyst 5.0/6.0^91^ or Prism version 10. The use of n was used in the results to refer to the number of participants in a group and the number of metabolites being referred to in the text. Differences in demographic and socioeconomic data between the groups (healthy BMI (≤25) vs. overweight/obese BMI (>25) and LD vs. NLD) were analyzed using unpaired t-tests for continuous variables and Fisher’s exact test for categorical variables. Details of statistical analyses used can be found in the figure and table legends.

## Supplemental information

Document S1. Figures S1–S5 and Tables S1–S3

Table S4. Excel file containing additional data too large to fit in a PDF, related to Figure 3

Table S5. Excel file containing additional data too large to fit in a PDF, related to Figure 3

Table S6. Excel file containing additional data too large to fit in a PDF, related to Figure 3
