# Supplementary material for: Metabolic comparison of minimally to noninvasive urogenital sample types for studying gynecologic health: A pilot study
Source: iScience. 2025 Jun 18;28(7):112938. doi: 10.1016/j.isci.2025.112938 (PMC12270662; doi:10.1016/j.isci.2025.112938)
Supplement: Document S1. Figures S1–S5 and Tables S1–S3 [file mmc1.pdf]

iScience, Volume *28*

Supplemental information

Metabolic comparison of minimally to noninvasive urogenital sample types for studying gynecologic health: A pilot study

Holly Chatenoud, Paweł Łaniewski, Nichole D. Mahnert, and Melissa M. Herbst-Kralovetz

Figure S1. Greater *Lactobacillus crispatus* abundance in *Lactobacillus* dominant (LD) group compared to non-*Lactobacillus* dominant (NLD) group, related to Table 1. Percentage relative abundance of the most abundant vaginal taxa at species level >10,000 counts, between *Lactobacillus* dominant (LD) and non-*Lactobacillus* dominant (NLD) groupings. Analysis was performed using MicrobiomeAnalyst 2.0.

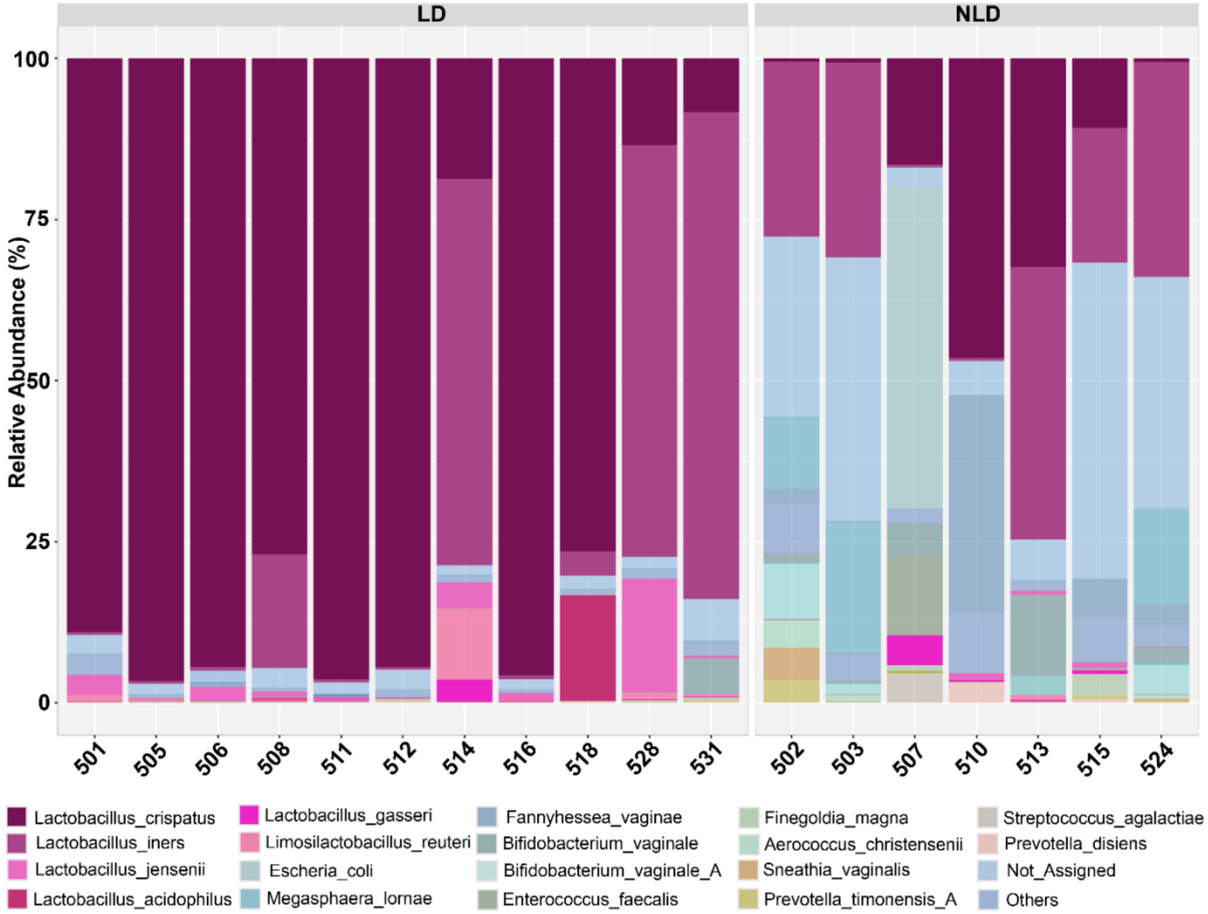

Figure S2. Metabolites contributing to the separation between urogenital sample types in PLSDA, related to Figure 1. Top 15 metabolites contributing to components one and two used to construct the PLSDA plot in Figure 1. Superpathways of the metabolites are color coded. Metabolites are also color coded according to if they were identified (green) or unidentified (yellow) in each sample type.

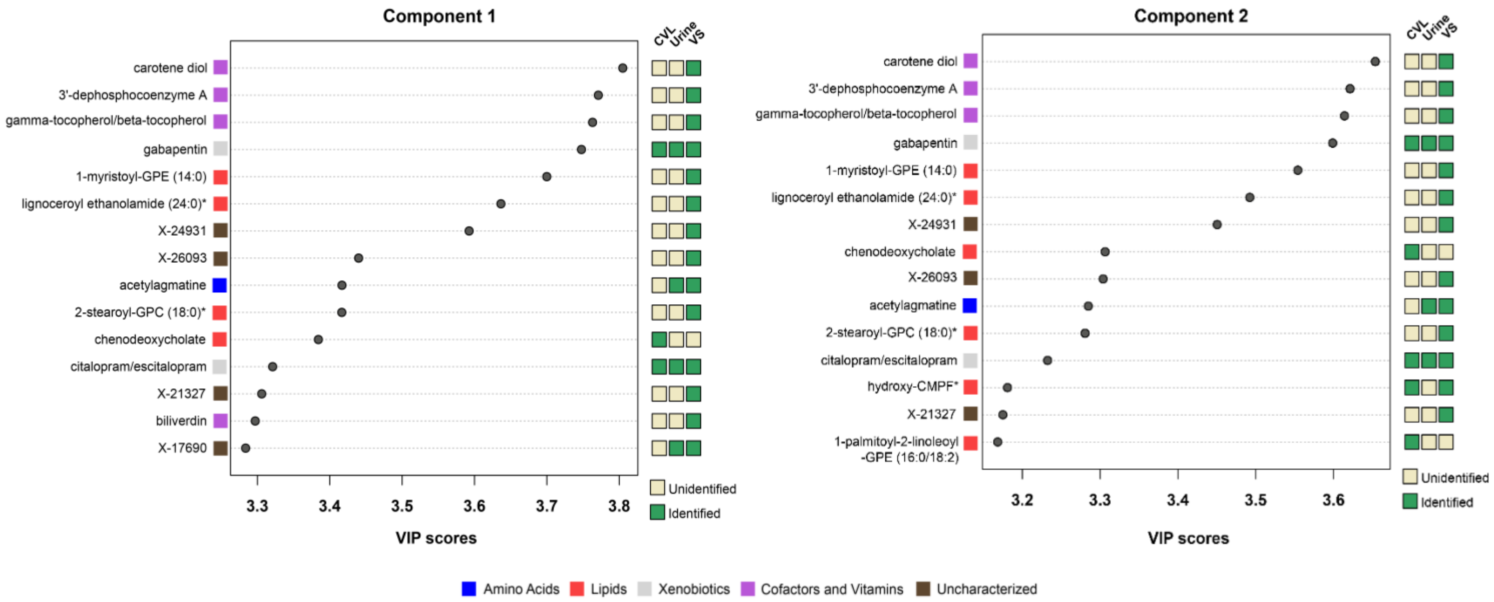

Figure S3. FLOQswab controls exhibit different global metabolic profiles to vaginal swab samples, related to Figure 1. Blank FLOQswabs were utilized as controls to test for false positive metabolic signatures observed in the vaginal swab sample types. Different metabolic profiles were exhibited in the vaginal swab samples and the blank controls, indicating the swab itself did not influence the metabolic profiles detected by the vaginal swabs.

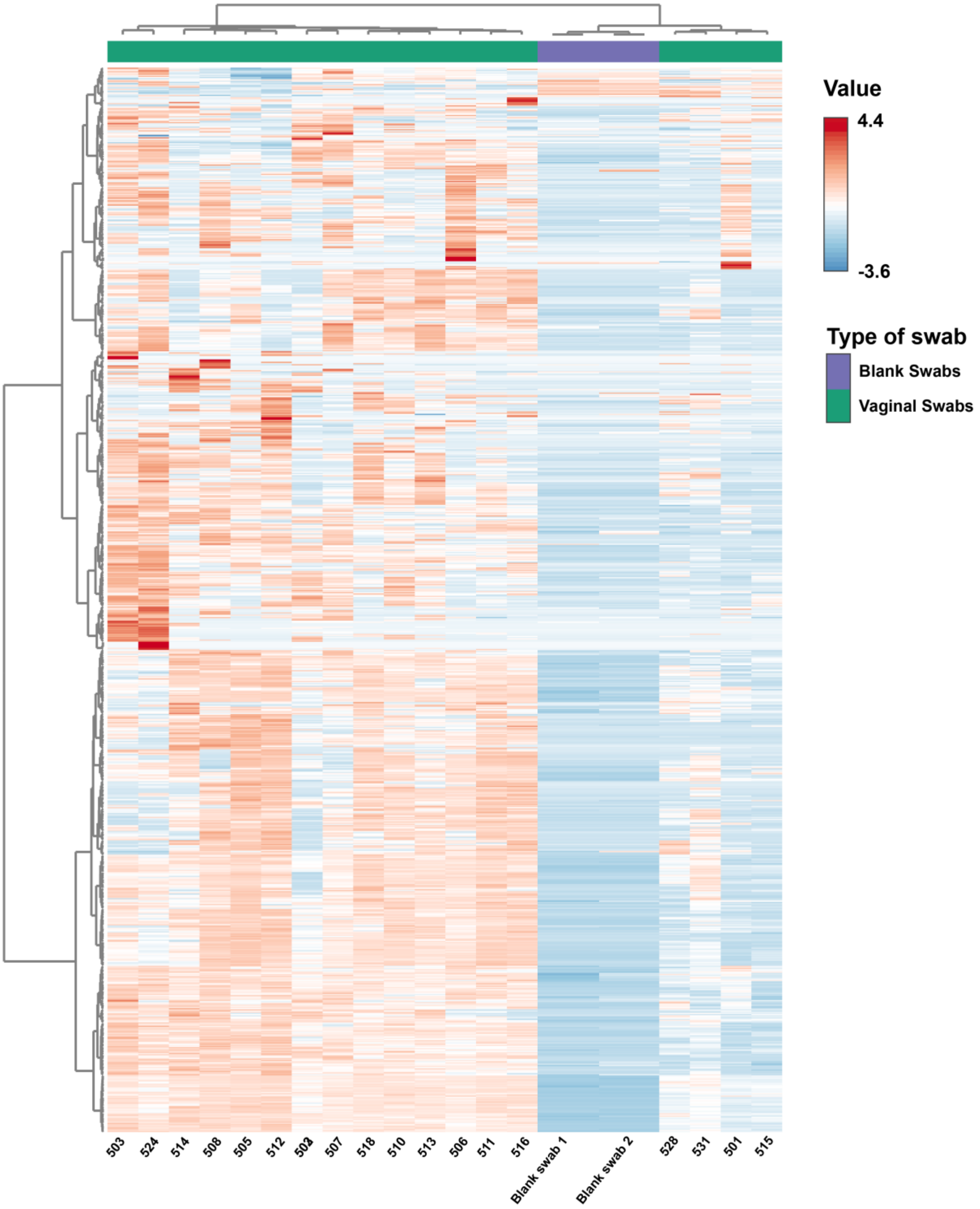

Figure S4. Lactobacillus dominance grouped relative abundance data without log-transformation, related to Figure 4. The batch-normalised metabolomics data is used to present the relative abundance of key metabolites in individuals with *Lactobacillus* dominant (≥80%) and non-*Lactobacillus* dominant (<80%) samples. Shown are key metabolites identified in urine (A), CVL (B) and vaginal swab (C) samples. Significant differences in metabolite relative abundances between the groups were calculated using a non-parametric Mann-Whitney test. Actual *p*-values are shown in the figure.

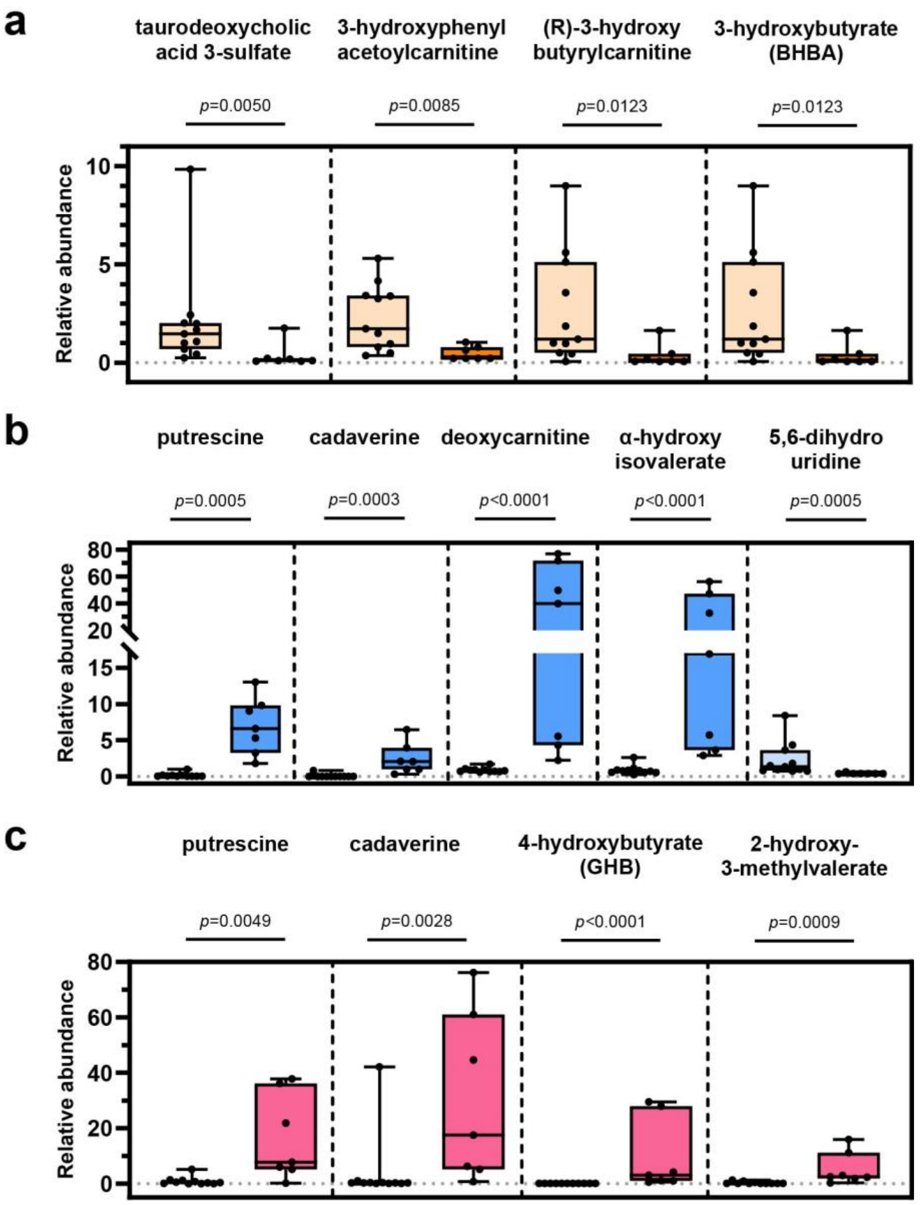

Figure S5. BMI grouped relative abundance data without log-transformation, related to Figure 5. The batch-normalised metabolomics data is used to present the relative abundance of key metabolites in individuals with a healthy BMI (≤25) and an overweight/obese BMI (>25). Shown are key metabolites identified in urine (A), CVL (B) and vaginal swab (C) samples. Significant differences in metabolite relative abundances between the groups were calculated using a non-parametric Mann-Whitney test. Actual *p*-values are shown in the figure.

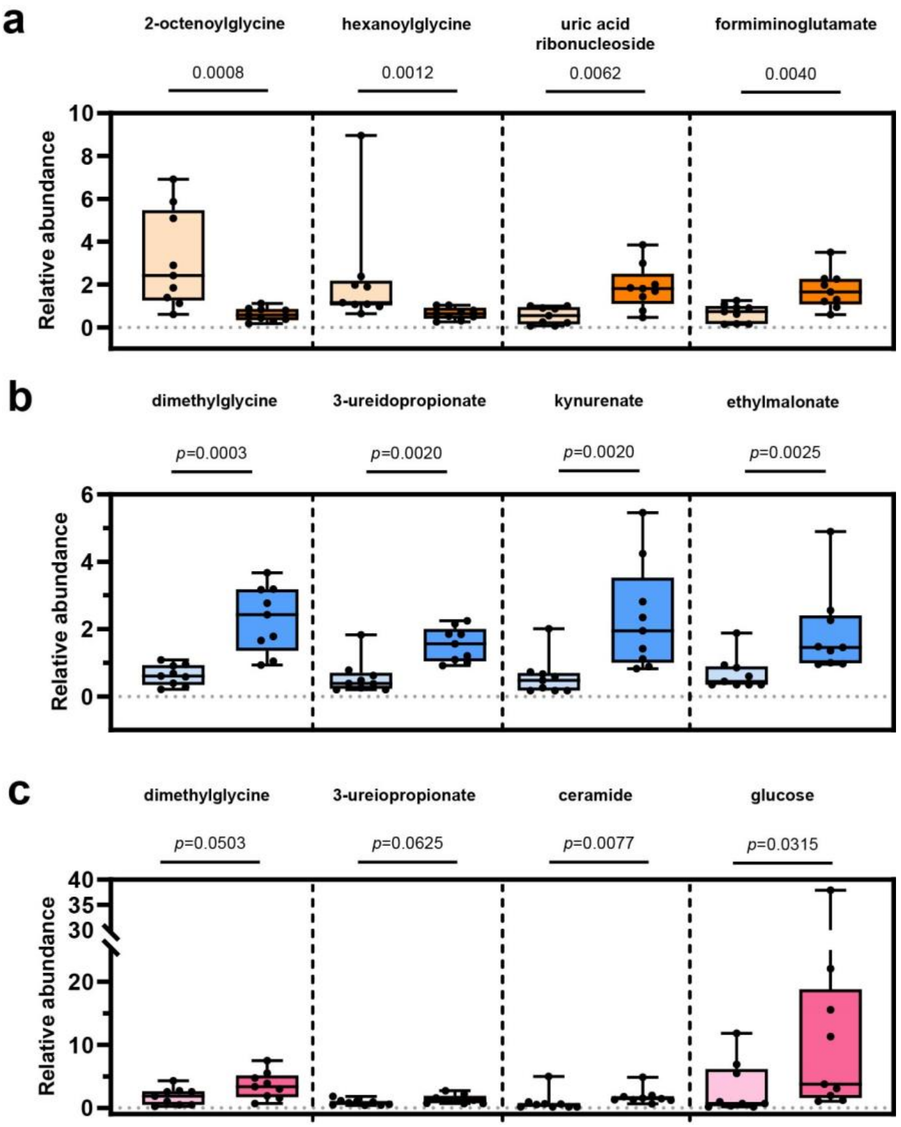

Table S1. Additional participant characteristics dichotomized by *Lactobacillus* dominance, related to Table 1. *p*-values were calculated using Fisher's exact test for categorical variables and unpaired t-test for continuous variables. Bold represents a significant *p*-value <0.05. [a] Recent use within 6 months of survey collection.

	All (n=18)	*Lactobacillus* dominant (>80%) (n=9)	Non-*Lactobacillus* dominant (<80%) (n=9)	*p*-value
**Contraceptive use[a] (n (%)) (n=18)**				
**Barrier Methods (n=16)**				
Yes	7 (43.75)	5 (50.00)	2 (33.33)	0.6329
No	9 (56.25)	5 (50.00)	4 (66.67)	
**Birth Control Pill (n=18)**				
Yes	3 (16.67)	2 (18.18)	1 (14.29)	>0.9999
No	15 (83.33)	9 (81.82)	6 (85.71)	
**Copper IUD (n=15)**				
Yes	4 (26.67)	3 (30.00)	1 (20.00)	>0.9999
No	11 (73.33)	7 (70.00)	4 (80.00)	
**Depo-Provera (n=18)**				
Yes	1 (5.56)	1 (9.09)	0 (0.00)	>0.9999
No	17 (94.44)	10 (90.91)	7 (100.00)	
**Progesterone IUD (n=18)**				
Yes	3 (16.67)	1 (9.09)	2 (28.57)	0.5282
No	15 (83.33)	10 (90.91)	5 (71.43)	
**Hormonal (n=12)**				
Yes	6 (50.00)	3 (37.50)	3 (75.00)	0.5455
No	6 (50.00)	5 (62.50)	1 (25.00)	
**Non-hormonal (n=12)**				
Yes	6 (50.00)	5 (62.50)	1 (25.00)	0.5455
No	6 (50.00)	3 (37.50)	3 (75.00)	
**Menstrual Products (n (%)) (n=17)**				
**Tampons**				
Yes	13 (72.22)	9 (81.82)	4 (57.14)	0.3260
No	5 (27.78)	2 (18.18)	3 (42.86)	
**Pads**				
Yes	11 (61.11)	9 (81.82)	2 (28.57)	**0.0491**
No	7 (38.89)	2 (18.18)	5 (71.43)	
**Cervical cup**				
Yes	3 (16.67)	1 (9.09)	2 (28.57)	0.5282
No	15 (83.33)	10 (90.91)	5 (71.43)	
**Douching[a] (n (%)) (n=17)**				
Yes	2 (11.11)	1 (9.09)	1 (14.29)	>0.9999
No	16 (88.89)	10 (90.91)	6 (85.71)	
**Antibiotic use[a] (n (%)) (n=17)**				
Yes	3 (20.00)	2 (25.00)	1 (14.29)	>0.9999
No	15 (80.00)	6 (75.00)	6 (85.71)	
**Perceived Stress Score (mean (S.D.)) (n=17)**	15.50 (4.80)	15.73 (4.18)	12.86 (5.17)	0.2403

Table S2. Additional participant characteristics dichotomized by BMI, related to Table 2. *p*-values were calculated using Fisher's exact test for categorical variables and unpaired t-test for continuous variables. [a] Recent use within 6 months of survey collection.

	All (n=18)	BMI ≤25 (n=9)	BMI >25 (n=9)	*p*-value
**Contraceptive use[a] (n (%)) (n=18)**				
**Barrier Methods (n=16)**				
Yes	7 (43.75)	5 (55.56)	2 (28.57)	0.3575
No	9 (56.25)	4 (44.44)	5 (71.43)	
**Birth Control Pill (n=18)**				
Yes	3 (16.67)	1 (11.11)	2 (22.22)	>0.9999
No	15 (83.33)	8 (88.89)	7 (77.78)	
**Copper IUD (n=15)**				
Yes	4 (26.67)	3 (33.33)	1 (16.67)	0.6044
No	11 (73.33)	6 (66.67)	5 (83.33)	
**Depo-Provera (n=18)**				
Yes	1 (5.56)	0 (0.00)	1 (11.11)	>0.9999
No	17 (94.44)	9 (100.00)	8 (88.89)	
**Progesterone IUD (n=18)**				
Yes	3 (16.67)	0 (0.00)	3 (33.33)	0.2059
No	15 (83.33)	9 (100.00)	6 (66.67)	
**Hormonal (n=12)**				
Yes	6 (50.00)	1 (16.67)	5 (83.33)	0.0801
No	6 (50.00)	5 (83.33)	1 (16.67)	
**Non-hormonal (n=12)**				
Yes	6 (50.00)	5 (83.33)	1 (16.67)	0.0801
No	6 (50.00)	1 (16.67)	5 (83.33)	
**Menstrual Products (n (%)) (n=17)**				
**Tampons**				
Yes	13 (72.22)	8 (88.89)	5 (55.56)	0.2941
No	5 (27.78)	1 (11.11)	4 (44.44)	
**Pads**				
Yes	11 (61.11)	6 (66.67)	5 (55.56)	>0.9999
No	7 (38.89)	3 (33.33)	4 (44.44)	
**Cervical cup**				
Yes	3 (16.67)	2 (22.22)	1 (11.11)	>0.9999
No	15 (83.33)	7 (77.78)	8 (88.89)	
**Douching[a] (n (%)) (n=17)**				
Yes	2 (11.11)	0 (0.00)	2 (22.22)	0.4706
No	16 (88.89)	9 (100.00)	7 (77.78)	
**Antibiotic use[a] (n (%)) (n=17)**				
Yes	3 (20.00)	0 (0.00)	3 (37.50)	0.2088
No	15 (80.00)	6 (100.00)	5 (62.50)	
**Perceived Stress Score (mean (SD)) (n=17)**	15.50 (4.80)	14.56 (4.03)	14.67 (5.46)	0.9636

Table S3. Composition of superpathways and subpathways of metabolites identified in three urogenital biospecimens, related to Figure 2.

	Urine (n, (%))	CVLs (n, (%))	Vaginal swabs (n, (%))
**Superpathways**	**N = 1569**	**N = 615**	**N = 947**
Amino acids	292, (18.6)	187, (30.4)	206, (25.6)
Lipids	210, (13.4)	119, (19.3)	288, (35.8)
Xenobiotics	301, (19.2)	93, (15.1)	121, (15.0)
Peptides	52, (3.31)	44, (7.15)	53, (5.60)
Nucleotides	56, (3.57)	40, (6.50)	55, (5.81)
Carbohydrates	39, (2.49)	32, (5.20)	38, (4.01)
Vitamins and cofactors	49, (3.12)	22, (3.58)	33, (3.48)
Energy	17, (1.08)	12, (1.95)	11, (1.16)
Partially Characterized	64, (4.07)	6, (0.98)	9, (0.95)
Uncharacterized	489, (31.2)	60, (9.76)	133, (14.0)
**Lipid subpathways**	**N = 210**	**N = 119**	**N = 288**
Ceramides	0, (0.00)	5, (4.20)	18, (6.25)
Fatty acids	40, (19.1)	16, (13.5)	54, (18.8)
Glycerolipids	2, (0.95)	4, (3.36)	27, (9.38)
Glycerophospholipids	18, (8.57)	38, (31.9)	60, (20.8)
Other Fatty Acids	53, (25.2)	21, (17.7)	33, (11.5)
Sphingolipids	13, (6.19)	20, (16.8)	82, (28.5)
Sterol lipids	84, (40.00)	15, (12.6)	14, (4.86)
**Amino Acid subpathways**	**N = 292**	**N = 187**	**N = 206**
Alanine and Aspartate metabolism	10, (3.42)	9, (4.81)	8, (3.88)
Creatine metabolism	5, (1.71)	3, (1.60)	4, (1.94)
Glutamate metabolism	16, (5.48)	9, (4.81)	7, (3.40)
Glutathione metabolism	6, (2.05)	8, (4.28)	8, (3.88)
Glycine, serine, and threonine metabolism	13, (4.45)	10, (5.35)	10, (4.85)
Guanidino and acetamido metabolism	3, (1.03)	1, (0.53)	2, (0.97)
Histidine Metabolism	24, (8.22)	22, (11.8)	23, (11.2)
Lactoyl amino acid metabolism	6, (2.05)	6, (3.21)	6, (2.91)
Lysine metabolism	27, (9.25)	17, (9.09)	20, (9.71)

	Urine (n, (%))	CVLs (n, (%))	Vaginal swabs (n, (%))
**Amino Acid subpathways**	**N = 292**	**N = 187**	**N = 206**
Leucine, isoleucine and valine metabolism	39, (13.4)	21, (11.2)	28, (13.6)
Methionine, cysteine, SAM and taurine metabolism	25, (8.56)	20, (10.7)	23, (11.2)
Phenylalanine metabolism	9, (3.08)	5, (2.67)	7, (3.40)
Polyamine metabolism	12, (4.11)	11, (5.88)	14, (6.80)
Tryptophan metabolism	34, (11.6)	12, (6.42)	14, (6.80)
Tyrosine metabolism	34, (11.6)	11, (5.88)	10, (4.85)
Urea cycle; arginine and proline metabolism	29, (9.93)	22, (11.8)	22, (10.7)
**Xenobiotics**	**N = 301**	**N = 93**	**N = 121**
Bacterial/fungal	5, (1.66)	1, (1.08)	2, (1.65)
Benzoate metabolism	47, (15.6)	18, (19.4)	23, (19.0)
Chemical	46, (15.3)	12, (12.9)	16, (13.2)
Drug- Analgesics and anesthetics	17, (5.65)	11, (11.8)	7, (5.79)
Drug – Antibiotics	1, (0.33)	0, (0.00)	0, (0.00)
Drug - Cardiovascular	4, (1.33)	1, (1.08)	1, (0.83)
Drug – Metabolic	1, (0.33)	1, (1.08)	1, (0.83)
Drug- Neurological	2, (0.66)	1, (1.08)	1, (0.83)
Drug - Other	1, (0.33)	0, (0.00)	0, (0.00)
Drug- Psychoactive	7, (2.33)	2, (2.15)	2, (1.65)
Drug - Respiratory	3, (1.00)	0, (0.00)	0, (0.00)
Drug – Topical agents	4, (1.33)	1, (1.08)	3, (2.48)
Food component/plant	140, (46.5)	32, (34.4)	48, (39.7)
Tobacco metabolite	7, (2.33)	3, (3.23)	3, (2.48)
Xanthine metabolism	16, (5.32)	10, (10.8)	14, (11.6)
